# Red Cell Distribution Width and Risk of Atrial Fibrillation and Subsequent Thromboembolism: The Tromsø Study

**DOI:** 10.1055/s-0040-1716417

**Published:** 2020-09-28

**Authors:** Erin M. Hald, Maja-Lisa Løchen, Jostein Lappegård, Trygve S. Ellingsen, Ellisiv B. Mathiesen, Tom Wilsgaard, Inger Njølstad, Sigrid K. Brækkan, John-Bjarne Hansen

**Affiliations:** 1 K.G. Jebsen Thrombosis Research and Expertise Center (TREC), Department of Clinical Medicine, UiT The Arctic University of Norway, Tromsø, Norway; 2 Division of Internal Medicine, University Hospital of North Norway, Tromsø, Norway.; 3 Epidemiology of Chronic Diseases Research Group, Department of Community Medicine, UiT The Arctic University of Norway, Tromsø, Norway.; 4 Brain and Circulation Research Group, Department of Clinical Medicine, UiT The Arctic University of Norway, Tromsø, Norway.

**Keywords:** venous thrombosis, arterial thrombosis, epidemiological studies, red cell distribution width

## Abstract

**Introduction** Red cell distribution width (RDW) is associated with cardiovascular diseases, including atrial fibrillation (AF) and venous thromboembolism (VTE). Whether RDW is a risk marker for thromboembolic events in AF patients is scarcely known. We aimed to assess the association between RDW and the risk of AF, and AF-related VTE and ischemic stroke, in a population-based cohort.

**Methods** We measured RDW in 26,111 participants from the Tromsø Study (1994–1995), and registered incident AF cases through December 31, 2013. Among participants with AF, first-ever VTEs and ischemic strokes were registered from the date of AF diagnosis through the end of follow-up. We calculated hazard ratios (HRs) with 95% confidence intervals (CIs) for AF by quartiles of RDW. Furthermore, we calculated cause-specific HRs for VTE and ischemic stroke by tertiles of RDW for participants with AF.

**Results** There were 2,081 incident AF cases during a median of 18.8 years of follow-up. Subjects with RDW in the highest quartile (RDW ≥ 13.3%) had 30% higher risk of AF than those in the lowest quartile (RDW ≤ 12.3%). Among those with AF, subjects with RDW in the upper tertile had a doubled risk of ischemic stroke (HR 2.07, 95% CI 1.20–3.57). In contrast, RDW was not associated with incident VTE in subjects with AF.

**Conclusion** RDW was significantly associated with incident AF in a general population. Among subjects with AF, high RDW was associated with ischemic stroke, but not VTE.

## Introduction

Atrial fibrillation (AF) is the most prevalent arrhythmia of clinical importance, and contributes extensively to morbidity and mortality in the population. 1 2 3 Ischemic stroke is generally considered the most detrimental complication of AF, 4 but recent studies have also established AF as a risk factor for venous thromboembolism (VTE). 5 6

Red cell distribution width (RDW) is an inexpensive and easily available measure of variation in erythrocyte size. Traditionally, RDW has been used in the differentiation of anemias, but RDW has recently also emerged as a risk marker for cardiovascular morbidity and mortality, 7 8 9 including myocardial infarction (MI), 9 heart failure, 10 ischemic stroke, 11 12 and VTE. 13 14 A few studies have also implicated RDW as a risk marker for AF. 15 16 17 18 In a case–control study including 117 patients with AF and 60 controls, RDW was found to be associated with AF in multivariable logistic regression analysis, 16 and in another case–control study by Liu et al, RDW was significantly higher in the AF group than in controls (12.7% vs. 12.4%; *p* < 0.05). 17 A recent meta-analysis examining the impact of hematological parameters on AF occurrence found higher RDW in participants with new-onset AF compared with those with sinus rhythm. 19 Furthermore, RDW was significantly increased in patients with AF recurrence. 19

To what extent RDW is associated with thromboembolic outcomes in patients with AF has not been extensively studied. A dose–response relationship between increasing RDW and risk of stroke in AF patients was found in a large registry-based study, 20 while small case–control studies have reported diverging results. 21 22 Interestingly, increasing CHA2DS2-VASC score, an established prediction score for thromboembolism in AF patients, 23 24 was positively correlated with RDW levels in subjects with AF in several studies. 17 20 25 Whether RDW contributes to the excess VTE risk in patients with AF is not known.

In the present study, we aimed to assess (1) the association between RDW and incident AF in a general population cohort, and (2) whether RDW was associated with incident thromboembolism (VTE and ischemic stroke) in subjects with AF.

## Methods

### Study Population

Participants were recruited from the fourth survey of the Tromsø Study, an ongoing, prospective health study of the inhabitants of Tromsø, Norway. The fourth survey was performed in 1994 to 1995, and all inhabitants in the Tromsø municipality aged ≥ 25 years were invited to participate. In total, 27,158 persons aged 25 to 97 years attended the study, comprising 77% of the eligible population. We excluded those who withdrew their consent to medical research after the Tromsø Study inclusion date ( *n* = 166), participants not officially registered as residents of Tromsø ( *n* = 22), participants with AF prior to the inclusion date ( *n* = 234), and persons with missing RDW measurements ( *n* = 625). A total of 26,111 participants were included in the study and followed from the date of enrolment to December 31, 2013. Incident cases of AF during follow-up were registered. In secondary analyses, persons with VTE ( *n* = 82) or ischemic stroke ( *n* = 200) prior to AF diagnosis were excluded, and VTE and ischemic stroke events were recorded in all persons with a first lifetime diagnosis of AF ( *n* = 1,812) from the day of AF diagnosis until the end of follow-up. All participants provided informed, written consent, and the Regional Committee for Medical and Health Research Ethics approved the study.

### Baseline Measurements

Nonfasting blood samples, self-administered questionnaires, and a physical examination were used to obtain baseline information on all study participants. For the blood cell parameters measurements, including RDW, 5 mL of blood was drawn from an antecubital vein into a vacutainer containing EDTA as an anticoagulant, and analyzed within 12 hours in an automated blood cell counter (Coulter Counter, Coulter Electronics, Luton, United Kingdom). RDW was calculated by dividing the standard deviation of mean corpuscular volume (MCV) by each person's MCV value and multiplying by 100 to convert to a percentage. 26 The analytic coefficient of variation of the RDW measurements was less than 3%. All blood samples were taken at the inclusion date (in 1994/1995). Serum total cholesterol was measured as previously described. 27 Blood pressure measurements were performed using an automatic device (Dinamap Vital Signs Monitor, 1846; Critikon Inc., Tampa, Florida, United States). After 2 minutes seated rest, three recordings were taken on the upper right arm at intervals of 1 minute, and the mean of the last two values was used in the analyses. Participants with systolic blood pressure ≥ 140 mm Hg, diastolic blood pressure ≥ 90 mm Hg, or currently using antihypertensive drugs were classified as having hypertension. Height and weight were measured with the participant in light clothing and no shoes. Body mass index (BMI) was calculated as weight in kilograms divided by the height in meters squared (kg/m ^2^ ). Self-administered questionnaires were used to obtain information on diabetes mellitus and current smoking status. Information on prior MI was obtained from the cardiovascular outcome registry of the Tromsø Study.

### Ascertainment of Atrial Fibrillation

Incident AF was identified by searching the discharge diagnosis registry at the University Hospital of North Norway, the sole provider of hospital care for the entire Tromsø municipality, and the Norwegian Cause of Death Registry provided by the Norwegian Institute of Public Health. The Tromsø Study participants' unique identification numbers were linked to these diagnostic registries using the International Classification of Diseases, Ninth Revision (ICD-9) codes 427.0 to 427.99 and Tenth Revision (ICD-10) codes I47 and I48. For subjects with a diagnosis of cardiovascular or cerebrovascular disease, but without a registered arrhythmia diagnosis, paper versions of hospital records (used until 2001) were manually searched for any mention of AF, and the term “atrial fibrillation” was used for text searches in the electronic records. Trained personnel reviewed each potential AF case's medical record. All definite AF diagnoses required electrocardiogram documentation, and were adjudicated by an independent endpoint committee. When possible, the AF events were further classified into paroxysmal and persistent versus permanent forms. Participants who had paroxysmal AF initially, but who eventually developed a permanent form, were classified as having permanent AF. Persons with transient AF occurring only in relation to cardiac surgery or an acute MI, and subjects who only had AF in the terminal phase (the last week) of life were not classified as having AF. 28

### Ascertainment of Venous Thromboembolism

We identified all incident VTE events by searching the hospital discharge diagnosis registry, the autopsy registry, and the radiology procedure registry at the University Hospital of North Norway as previously described in detail. 29 Trained personnel reviewed the medical records of each potential VTE event for case validation. A VTE episode was recorded as a validated outcome only when clinical VTE symptoms were present and combined with confirmatory tests (compression ultrasonography, venography, spiral computed tomography, perfusion–ventilation scan, pulmonary angiography, or autopsy), resulting in a VTE diagnosis that required treatment. For the VTE events retrieved from autopsy records, a validated event was recorded when the death certificate designated VTE as the cause of death or a significant condition contributing to death. The VTE event was further classified as unprovoked (no provoking factors) or provoked (≥ one provoking factor[s]) based on the presence of provoking factors at the time of diagnosis. Immobilization (bed rest ≥ 3 days, wheelchair, long haul travel ≥ 4 hours within 14 days prior to the event), major surgery, trauma, or an acute medical condition (acute MI, ischemic stroke, or major infectious disease) within 8 weeks prior to the event, active cancer, or other potential provoking factors described by a physician in the medical record (e.g., intravascular catheter) were regarded as provoking factors.

### Ascertainment of Ischemic Stroke

Information on incident ischemic stroke was obtained by linkage to the diagnosis registries at the University Hospital of North Norway and the Norwegian Cause of Death Registry, using an expansive search for the ICD-8 and -9 codes 430–438, and ICD-10 codes I60–I69 (cerebrovascular diseases). Systematic text searches in the medical records for patients with ICD-8 and -9 diagnosis codes 410–414 and 798–799, and ICD-10 codes I20–I25 and R96, R98, and R99 were additionally performed to ensure case completeness. The WHO definition was used to define ischemic stroke: Rapidly developing clinical signs of focal or global disturbance of cerebral function, with symptoms lasting 24 hours or longer or leading to death, with no apparent cause other than vascular origin. 30 Furthermore, imaging tests (computed tomography or magnetic resonance imaging) or an autopsy were required to exclude intracerebral or subarachnoid hemorrhage. An independent endpoint committee followed a detailed protocol according to established diagnostic criteria for case validation.

### Statistical Analyses

We performed statistical analyses using STATA, version 15 (Stata Corporation, College Station, Texas, United States). For analysis of the association between RDW and AF, crude incidence rates (IRs) with 95% confidence intervals (CIs) were calculated as the total number of events divided by total person-time at risk, and expressed as events per 1,000 person-years. Cox proportional hazards regression models were used to estimate hazard ratios (HRs) with 95% CI for AF by increasing levels of RDW. To assess for nonlinearity or a potential threshold effect, participants were categorized into quartiles based on the distribution of baseline RDW, and the lowest quartile was used as reference. Additionally, a cutoff was fixed at the 95th percentile. Model 1 included age, sex, and BMI, model 2 included the variables of model 1, as well as hemoglobin, white blood cells, and platelet count, and model 3 included the variables of model 2, as well as smoking, hypertension, diabetes, previous MI, and total cholesterol. We used chronological age as the time scale in the regression models, defining the participants' age at study enrollment as entry time, and the age at the time of AF diagnosis or censoring event (i.e., migration, death, or study end) as exit time.

In analyses regarding the risk of VTE and ischemic stroke by RDW levels in the AF population, tertiles based on the baseline RDW distribution were chosen for the categorical analyses due to the smaller population size. Cause-specific Cox proportional hazards models were used to estimate HRs with 95% CIs for VTE and ischemic stroke. In the cause-specific models, the AF patients were followed to the first occurrence of VTE or stroke (e.g., in the analyses of stroke risk, a participant who developed VTE before a stroke would be censored at the date of VTE). Age at AF diagnosis was used as entry time, and the age at the time of a censoring event (i.e., VTE, ischemic stroke, migration, death, or study end) as exit time. The number of participants included in the adjusted regression models varied slightly due to missing data for some covariates (in total 1.5% missing). We tested the assumption of proportional hazards assumption using Schoenfeld residuals and found no violation. Statistical interactions between RDW and sex were tested by including cross-product terms of sex and RDW into the models, and no interactions were found.

## Results

A total of 2,082 participants (8.0%) experienced incident AF during a median follow-up time of 18.8 years, yielding a crude IR of 4.7 per 1,000 person-years. The mean age at AF diagnosis was 75.0 years (range 34.3–103.1 years). The mean RDW of the study population was 12.9% with a standard deviation of 0.93. Baseline characteristics of study participants across quartiles of RDW are shown in Table 1 . The mean age at study inclusion was 12.5 years higher for participants in the highest versus the lowest quartile of RDW. White blood cell count, BMI, total cholesterol, and the prevalence of daily smoking, diabetes, prior MI, and hypertension increased across quartiles of RDW ( Table 1 ). Participants who developed AF during follow-up were older, had higher BMI, and total cholesterol levels, and a higher prevalence of diabetes and prior MI compared with participants without AF ( Table 2 ). Hemoglobin levels and white blood cell counts were higher in those with AF, while there were more daily smokers among participants without than with AF ( Table 2 ).

**Table 1:** Baseline characteristics of study participants by quartiles of red cell distribution width (the Tromsø Study 1994–2013)

RDW	Quartile 1 ( *n* = 6,549)	Quartile 2 ( *n* = 6,682)	Quartile 3 ( *n* = 6,613)	Quartile 4 ( *n* = 6,267)
RDW range (%)	10.7–12.3	12.4–12.7	12.8–13.2	13.3–30.5
Age (y)	40.7 ± 12.3	44.6 ± 13.8	48.6 ± 14.7	53.2 ± 15.9
Sex (male, %)	46.1 (3,022)	49.0 (3,271)	49.5 (3,274)	44.2 (2,771)
Hemoglobin (g/dL)	14.1 ± 1.1	14.2 ± 1.1	14.1 ± 1.1	13.7 ± 1.4
White blood cells (× 10 ^9/^ L)	6.9 ± 1.8	7.0 ± 2.1	7.1 ± 2.0	7.3 ± 2.2
Platelets (× 10 ^9^ /L)	251 ± 51	249 ± 52	251 ± 55	260 ± 66
Body mass index (kg/m ^2^ )	24.7 ± 3.5	25.1 ± 3.7	25.3 ± 3.9	25.5 ± 4.2
Total cholesterol (mmol/L)	5.7 ± 1.2	6.0 ± 1.3	6.2 ± 1.3	6.3 ± 1.4
Diabetes (%)	1.4 (88)	1.6 (105)	1.6 (128)	1.9 (121)
History of myocardial infarction (%)	4.4 (290)	7.3 (489)	11.2 (739)	15.9 (994)
Hypertension a (%)	26.7 (1,745)	32.6 (2,179)	37.3 (2,469)	45.6 (2,860)

Abbreviation: RDW, red cell distribution width.

Note: Values are given as percentages with numbers in brackets or as means with standard deviations.

a Defined as systolic blood pressure ≥ 140 mm Hg or diastolic blood pressure ≥ 90 mm Hg or self-reported use of antihypertensive medication.

**Table 2:** Baseline characteristics by development of atrial fibrillation (the Tromsø Study 1994–2013)

	Without AF ( *n* = 25,029)	With AF ( *n* = 2,082)
RDW (%)	12.9 ± 0.9	13.1 ± 0.9
Age (y)	45.3 ± 14.4	62.7 ± 11.8
Sex (male, %)	46.6 (11,205)	54.4 (1,133)
Body mass index (kg/m ^2^ )	25.0 ± 3.8	26.9 ± 4.3
Total cholesterol (mmol/L)	6.0 ± 1.3	6.7 ± 1.2
Hypertension a (%)	32.5 (7,804)	69.6 (1,449)
Self-reported diabetes (%)	1.4 (344)	4.7 (98)
Daily smoking (%)	37.8 (9,070)	27.9 (579)
History of myocardial infarction (%)	7.7 (1,861)	31.3 (651)
Hemoglobin (total) (g/dL)	14.0 ± 1.2	14.3 ± 1.1
White blood cells (× 10 ^9/^ L)	7.1 ± 2.0	7.0 ± 2.0
Platelets, ×10 ^9^ /L	254 ± 56	242 ± 56

Abbreviations: AF, atrial fibrillation; RDW, red cell distribution width.

Note: Values are given as percentages with numbers in brackets or as means with standard deviations.

a Defined as systolic blood pressure ≥ 140 mm Hg or diastolic blood pressure ≥ 90 mm Hg or self-reported use of antihypertensive medication.

Crude IRs and HRs for AF across quartiles of RDW are shown in Table 3 . Participants with RDW in the highest quartile had 32% increased risk of incident AF compared with those in the lowest quartile in analyses adjusted for age, sex, and BMI (HR 1.32, 95% CI 1.14–1.52). The relative risk estimates remained unchanged after further adjustment for hematological parameters (model 2), and barely altered after addition of cardiovascular risk factors to the regression model (model 3) ( Table 3 ). Given the wide range of RDW in the highest quartile (13.3–30.5), we performed sensitivity analyses excluding participants with RDW measurements above the 95th percentile (≥ 14.4%), and found similar risk estimates as for the full cohort (data not shown). Participants with RDW measurements above the 95th percentile (≥ 14.4%) had a similar 1.3-fold increased VTE risk when compared with participants in the lowest quartile (multivariable HR 1.30, 95% CI 1.04–1.63) ( Table 3 ). When modeling RDW as a continuous variable, a 1% increment in RDW was associated with a 7% increased risk of AF (HR 1.07, 95% CI 1.02–1.12) after multivariable adjustment ( Table 3 ). We also performed age-stratified analyses in which the risk of AF was assessed in participants younger than and older than 60 years of age at inclusion. When comparing those in the highest versus the lowest quartile of RDW, the positive association was slightly more pronounced in the youngest age group, but the CIs overlapped (multivariable HR 1.38 [95% CI 1.11–1.74] and 1.27 [95% CI 1.09–1.49] for those ≤ 60 years and those < 60 years, respectively) (data not shown). To investigate whether the association between RDW and AF was modified by anemia, we performed analyses in which women with hemoglobin levels < 12 g/L ( *n* = 976) and men with hemoglobin levels < 13 g/L ( *n* = 338) were excluded. In analyses adjusted for BMI and sex, the risk estimates for nonanemic participants in the highest RDW quartile did not differ from that observed for the total population (HR 1.33, 95% CI 1.15–1.54) (data not shown).

**Table 3:** Crude incidence rates per 1,000 person‐years and adjusted hazard ratios with 95% confidence intervals for atrial fibrillation by quartiles of red cell distribution width (the Tromsø Study 1994–2013)

RDW	Persons	Median RDW, % (range)	Person-years	Events	IR	HR, model 1 a	HR, model 2 b	HR, model 3 c
Quartile 1	6,549	12.0 (10.7–12.3)	118,670	255	2.1 (1.9–2.4)	Ref	Ref	Ref
Quartile 2	6,682	12.6 (12.4–12.7)	117,361	446	3.8 (3.5–4.2)	1.14 (0.97–1.33)	1.13 (0.97–1.32)	1.12 (0.96–1.31)
Quartile 3	6,613	13.0 (12.8–13.2)	111,012	619	5.6 (5.2–6.0)	1.18 (1.02–1.37)	1.18 (1.02–1.37)	1.15 (0.99–1.33)
Quartile 4	6,267	14.1 (13.3–30.5)	95,056	762	8.0 (7.5–8.6)	1.32 (1.14–1.52)	1.32 (1.14–1.53)	1.27 (1.09–1.47)
> 95th percentile	1,317	15.7 (14.4–30.5)	18,771	138	7.4 (6.2–8.7)	1.36 (1.10–1.68)	1.49 (1.16–1.80)	1.30 (1.04–1.63)
HR per 1% increase in RDW						1.08 (1.03–1.12)	1.10 (1.05–1.15)	1.07 (1.02–1.12)

Abbreviations: IR, incidence rate; HR, hazard ratio; RDW, red cell distribution width.

a Age as time scale, adjusted for sex and body mass index.

b Age as time scale, adjusted for sex, body mass index, hemoglobin, white blood cells, and platelets.

c Age as time scale, adjusted for sex, body mass index, hemoglobin, white blood cells, platelets, smoking, hypertension, self-reported diabetes, previous myocardial infarction, and total cholesterol.

Among the 2,082 participants who developed AF during follow-up, 270 persons had an ischemic stroke ( *n* = 190) or VTE ( *n* = 80) before the AF diagnosis, and were excluded from the cause-specific analyses of RDW as a risk marker for AF-related thromboembolism. The remaining 1,812 participants with AF were followed for a median of 3.7 years, and there were 264 incident ischemic strokes and 87 VTEs during follow-up. Among the VTE events, 53 (61%) were pulmonary embolisms (PEs) and 34 (39%) were deep vein thromboses (DVTs). Fifty-eight (66.7%) of the VTE events had one or more provoking factor present at the time of diagnosis, and among the provoked events, 50% were attributed to an acute medical condition within 8 weeks prior to the VTE. Seventeen participants experienced both ischemic stroke and VTE during follow-up, and for these participants, follow-up ended when their first thromboembolic event occurred (VTE first = 5, stroke first = 12). The calculated crude IRs and HRs for VTE and ischemic stroke across tertiles of RDW are shown in Table 4 . Crude IRs for ischemic stroke were significantly higher in the highest tertile (IR 4.00 per 100 person-years, 95% CI 3.26–4.90) versus the lowest tertile (IR 2.30 per 100 person-years, 95% CI 1.84–2.89) of RDW. Furthermore, participants with RDW measurements in the highest tertile had a 51% increased risk of ischemic stroke compared with those in the lowest tertile after multivariable adjustment (HR 1.51, 95% CI 1.09–2.10) ( Table 4 ). On the other hand, there was no apparent increased risk of VTE by increasing RDW tertiles in any of the regression models ( Table 4 ). In subgroup analyses, RDW was not associated with either PE or DVT (data not shown).

**Table 4:** Crude incidence rates per 100 person-years and hazard ratios for incident venous thromboembolism and ischemic stroke by red cell distribution width in subjects with atrial fibrillation (the Tromsø Study 1994–2013)

	Persons	Median RDW, % (range)	Person-years	Events	IR	HR, model 1 a	HR, model 2 b	HR, model 3 c
Venous thromboembolism								
Tertile 1	627	12.4 (11.3–12.7)	3,256	29	0.89 (0.62–1.28)	Ref	Ref	Ref
Tertile 2	645	13.0 (12.8–13.3)	3,161	23	0.72 (0.48–1.09)	0.75 (0.43–1.31)	0.75 (0.43–1.30)	0.76 (0.43–1.34)
Tertile 3	540	14.1 (13.4–21.6)	2,328	23	0.99 (0.65–1.49)	1.01 (0.58–1.77)	1.00 (0.57–1.76)	0.97 (0.54–1.74)
> 95th percentile	93	15.6 (14.6–21.6)	368	2	0.54 (0.14–2.17)	0.56 (0.13–2.38)	0.57 (0.13–2.45)	0.58 (0.13–2.52)
HR per 1% increase in RDW						1.09 (0.83–1.41)	1.11 (0.84–1.47)	1.11 (0.83–1.48)
Ischemic stroke								
Tertile 1	627	12.4 (11.3–12.7)	3,256	75	2.30 (1.84–2.89)	Ref	Ref	Ref
Tertile 2	645	13.0 (12.8–13.3)	3,161	91	2.88 (2.34–3.54)	1.17 (0.86–1.58)	1.17 (0.86–1.59)	1.20 (0.88–1.64)
Tertile 3	540	14.1 (13.4–21.6)	2,328	93	4.00 (3.26–4.90)	1.50 (1.10–2.04)	1.48 (1.07–2.03)	1.51 (1.09–2.10)
> 95th percentile	93	15.6 (14.6–21.6)	368	20	5.44 (3.51–8.43)	2.11 (1.28–3.46)	2.00 (1.17–3.41)	2.07 (1.20–3.57)
HR per 1% increase in RDW						1.18 (1.03–1.34)	1.17 (1.02–1.35)	1.18 (1.02–1.36)

Abbreviations: IR, incidence rate; HR, hazard ratio; RDW, red cell distribution width.

a Age as time scale, adjusted for sex and body mass index.

b Age as time scale, adjusted for sex, body mass index, hemoglobin, white blood cells, and platelets.

c Age as time scale, adjusted for sex, body mass index, hemoglobin, white blood cells, platelets, smoking, hypertension, self-reported diabetes, previous myocardial infarction, and total cholesterol.

## Discussion

In this population-based cohort study, we confirmed an association between RDW and incident AF and expanded the current knowledge on the impact of RDW on the risk of thromboembolic events in AF patients. The risk of ischemic stroke, but not VTE, was higher with increasing RDW in AF patients.

Our finding of RDW as a risk marker for incident AF is in concurrence with previous reports. In a comparable cohort from the Malmö Diet and Cancer Study, 15 similar HRs for AF by RDW as those obtained in our study were reported. In their study, the highest quartile of RDW was associated with a 1.3-fold increased risk of AF after multivariable analyses, and a 1% increment in RDW was associated with an 8% increase in AF risk. 15 Similarly, Li et al found elevated RDW to be significantly associated with prevalent AF in a recently published Chinese cross-sectional study. 18 In a meta-analysis comprising 2,721 participants with AF, higher baseline RDW was associated with incident AF, but there was significant heterogeneity across the studies. 31

The biological mechanisms linking increasing RDW to AF are unclear. High levels of RDW may reflect oxidative stress and inflammation, as these factors shorten the life span of red blood cells and hamper bone marrow function, leading to a more heterogeneous erythrocyte population. 32 33 As AF is associated with both systemic inflammation and oxidative stress, 34 35 the association with RDW may partially be mediated through these factors. In the present study, we found that the association between RDW and AF was virtually unchanged after adjustment for white blood cell count, suggesting that other mechanisms than inflammation may be involved in the interplay between RDW and AF. This is in concurrence with a previous report from the Tromsø Study, in which the association between RDW and MI and ischemic stroke was only slightly attenuated after adjustments for C-reactive protein. 36

A direct effect of erythrocyte dysfunction on the myocardium may contribute to the development of AF. A heterogeneous erythrocyte population may have lower deformability and decreased oxygen-carrier capacity, 37 contributing to reduced myocardial oxygenation and cardiac dysfunction, 38 in turn triggering AF. An association between reduced erythrocyte deformability and cardiac arrhythmias has been demonstrated previously. 39 As addressed by a recent review, 40 anemia is associated with both AF development and increased RDW. 40 In the present study, risk estimates for AF were not significantly modified by adjusting for hemoglobin levels in the regression models, nor by excluding anemic participants from the multivariable analyses.

Previous observational studies, including reports from the Tromsø Study, have reported an increased risk of both VTE 13 14 and ischemic stroke 11 12 by RDW in the general population. Several mechanisms underlying these associations have been postulated, including inflammatory conditions, renal dysfunction, malnutrition, and oxidative damage. 41 Increased RDW has also been associated with decreased red blood cell deformability, 37 which increases erythrocyte aggregation, 42 and thus may trigger thrombosis. 43 A few studies have previously explored whether increasing RDW is associated with an excess risk of thromboembolism in AF patients. 20 44 45 46 In accordance with our results, Saliba et al found RDW to be associated with both absolute and relative risks of stroke in subjects with AF. 20 In their population-based registry study, the stroke risk was 33% higher among AF patients in the highest versus the lowest quartile of RDW. 20 Similarly, in a retrospective study of 5,082 patients with AF, RDW values ≥ 13.9% conferred a 1.7-fold increased risk of thromboembolic events (ischemic stroke and peripheral embolism) compared with RDW < 13.9%. 45 Although AF is a risk factor for incident VTE, 6 47 48 RDW measures did not affect VTE risk in the present AF cohort.

The mechanisms behind the differential impact of RDW measures on the two thromboembolic outcomes are not known. RDW measures correlate with the CHA2DS2-VASC score, 17 25 46 whose components ( C ongestive heart failure, H ypertension, A ge > 75, D iabetes mellitus, S troke/TIA/thromboembolism, V ascular disease, A ge 65–74, S ex [female]), are associated with stroke risk in AF patients. In contrast, neither hypertension, diabetes mellitus, nor female gender have been associated with VTE risk in prospective studies. 27 49 Thus, it is possible that the excess risk of stroke by RDW in AF patients to some extent reflects a burden of cardiovascular risk factors that have little impact on VTE risk. 50

The thrombosis potential model proposed by Rosendaal two decades ago emphasizes the multicausal nature of VTE. 51 The model illustrates how a VTE develops once sufficient risk factors have accumulated in a patient, and that VTE risk factors have synergistic effects. 51 We have previously demonstrated that the risk of PE in the first 6 months following AF diagnosis is 11-fold higher when compared with subjects without AF, largely exceeding the comparable risk estimates for ischemic stroke. 48 In the present study, we observed a majority of provoked VTEs among the AF patients, and one-third of all VTE had suffered an acute medical condition within 8 weeks prior to VTE diagnosis. In concurrence with the thrombosis potential model, it is plausible that the accumulated risk factors in the present population (high age, AF, hospitalizations, and comorbidities) dilute the additional impact of RDW on VTE risk.

Our study has several strengths, including the large number of participants recruited from a general population, and the prospective design with a mean follow-up of almost 19 years. The high attendance rate reduces the risk of selection bias, and the detailed validation of exposures and outcome ensures a clear temporality of events. Several limitations also merit attention. RDW was only measured at inclusion, and may have fluctuated over time. Unfortunately, repeated measurements of RDW are not available for the Tromsø Study population. Nevertheless, nondifferential misclassification of this kind generally leads to an underestimation of true associations. In an earlier report from the Tromsø Study, we found that risk estimates for VTE based on baseline measurements of cardiovascular risk factors (time-fixed analyses) in general corresponded well with risk estimated based on repeated measurements (time-varying analyses). 52 The true incidence of AF in our study may be underestimated, as many episodes of AF are asymptomatic. Furthermore, patients with AF exclusively treated in general practice are not included. We lacked information on heart failure and the use of antithrombotic medication. As the latter effectively reduces thrombosis risk in AF patients, it is possible that we underestimate the true risk of VTE and ischemic stroke by RDW in our AF population. Furthermore, not having information on heart failure makes it difficult to assess the implementation of RDW to existing prediction scores in AF. Among the AF patients, the number of VTE events was small, and our study may be underpowered to detect weak associations.

In conclusion, RDW showed an association with incident AF. In patients with AF, RDW further aggravated the risk of ischemic stroke, but not the risk of VTE.

## References

[1] Kannel W B Benjamin E J Status of the epidemiology of atrial fibrillation Med Clin North Am 2008 92 01 17 40, ix, ix 18060995 10.1016/j.mcna.2007.09.002 PMC2245891

[2] Ball J Carrington M J McMurray J J Stewart S Atrial fibrillation: profile and burden of an evolving epidemic in the 21st century Int J Cardiol 2013 167 05 1807 1824 23380698 10.1016/j.ijcard.2012.12.093

[3] Chugh S S Havmoeller R Narayanan K Worldwide epidemiology of atrial fibrillation: a Global Burden of Disease 2010 Study Circulation 2014 129 08 837 847 24345399 10.1161/CIRCULATIONAHA.113.005119 PMC4151302

[4] Ali A N Abdelhafiz A Clinical and economic implications of AF related stroke J Atr Fibrillation 2016 8 05 1279 27909470 10.4022/jafib.1279 PMC5089483

[5] Hald E M Enga K F Løchen M L Venous thromboembolism increases the risk of atrial fibrillation: the Tromso study J Am Heart Assoc 2014 3 01 e000483 24385452 10.1161/JAHA.113.000483 PMC3959677

[6] Wang C C Lin C L Wang G J Chang C T Sung F C Kao C H Atrial fibrillation associated with increased risk of venous thromboembolism. A population-based cohort study Thromb Haemost 2015 113 01 185 192 25318828 10.1160/TH14-05-0405

[7] Borné Y Smith J G Melander O Hedblad B Engström G Red cell distribution width and risk for first hospitalization due to heart failure: a population-based cohort study Eur J Heart Fail 2011 13 12 1355 1361 21940730 10.1093/eurjhf/hfr127

[8] Borné Y Smith J G Melander O Engström G Red cell distribution width in relation to incidence of coronary events and case fatality rates: a population-based cohort study Heart 2014 100 14 1119 1124 24760701 10.1136/heartjnl-2013-305028

[9] Skjelbakken T Lappegård J Ellingsen T S Red cell distribution width is associated with incident myocardial infarction in a general population: the Tromsø Study J Am Heart Assoc 2014 3 04 e001109 25134681 10.1161/JAHA.114.001109 PMC4310408

[10] Huang Y L Hu Z D Liu S J Prognostic value of red blood cell distribution width for patients with heart failure: a systematic review and meta-analysis of cohort studies PLoS One 2014 9 08 e104861 25133510 10.1371/journal.pone.0104861 PMC4136732

[11] Lappegård J Ellingsen T S Skjelbakken T Red cell distribution width is associated with future risk of incident stroke. The Tromsø Study Thromb Haemost 2016 115 01 126 134 26290352 10.1160/TH15-03-0234

[12] Feng G H Li H P Li Q L Fu Y Huang R B Red blood cell distribution width and ischaemic stroke Stroke Vasc Neurol 2017 2 03 172 175 28989807 10.1136/svn-2017-000071 PMC5628378

[13] Zöller B Melander O Svensson P Engström G Red cell distribution width and risk for venous thromboembolism: a population-based cohort study Thromb Res 2014 133 03 334 339 24393657 10.1016/j.thromres.2013.12.013

[14] Ellingsen T S Lappegård J Skjelbakken T Brækkan S K Hansen J B Red cell distribution width is associated with incident venous thromboembolism (VTE) and case-fatality after VTE in a general population Thromb Haemost 2015 113 01 193 200 25274492 10.1160/TH14-04-0335

[15] Adamsson Eryd S Borné Y Melander O Red blood cell distribution width is associated with incidence of atrial fibrillation J Intern Med 2014 275 01 84 92 24344999 10.1111/joim.12181

[16] Güngör B Özcan K S Erdinler İ Elevated levels of RDW is associated with non-valvular atrial fibrillation J Thromb Thrombolysis 2014 37 04 404 410 23821044 10.1007/s11239-013-0957-1

[17] Liu T Shao Q Korantzopoulos P Relation of red blood cell distribution width with CHADS _2_ and CHA _2_ DS _2_ -VASc score in Chinese patients with non-valvular atrial fibrillation Int J Cardiol 2017 228 861 864 27889552 10.1016/j.ijcard.2016.11.255

[18] Li H Gu Y Liu M The relationship between red blood cell distribution width and atrial fibrillation in Asian population: a cross-sectional study Pacing Clin Electrophysiol 2019 42 09 1197 1203 31397913 10.1111/pace.13776

[19] Weymann A Ali-Hasan-Al-Saegh S Sabashnikov A Prediction of new-onset and recurrent atrial fibrillation by complete blood count tests: a comprehensive systematic review with meta-analysis Med Sci Monit Basic Res 2017 23 179 222 28496093 10.12659/MSMBR.903320 PMC5439535

[20] Saliba W Barnett-Griness O Elias M Rennert G The association between red cell distribution width and stroke in patients with atrial fibrillation Am J Med 2015 128 02 192 e111–198 10.1016/j.amjmed.2014.09.020 25447618

[21] Ertaş G Sönmez O Turfan M Neutrophil/lymphocyte ratio is associated with thromboembolic stroke in patients with non-valvular atrial fibrillation J Neurol Sci 2013 324(1-2):49 52 23084070 10.1016/j.jns.2012.09.032

[22] Lee K H Park H W Cho J G Red cell distribution width as a novel predictor for clinical outcomes in patients with paroxysmal atrial fibrillation Europace 2015 17 02 ii83 ii88 26842121 10.1093/europace/euv210

[23] Lip G Y Nieuwlaat R Pisters R Lane D A Crijns H J Refining clinical risk stratification for predicting stroke and thromboembolism in atrial fibrillation using a novel risk factor-based approach: the Euro Heart Survey on Atrial Fibrillation Chest 2010 137 02 263 272 19762550 10.1378/chest.09-1584

[24] Kirchhof P Benussi S Kotecha D 2016 ESC Guidelines for the management of atrial fibrillation developed in collaboration with EACTS Europace 2016 18 11 1609 1678 27567465 10.1093/europace/euw295

[25] Kurt M Tanboga I H Buyukkaya E Karakas M F Akçay A B Sen N Relation of red cell distribution width with CHA2DS2-VASc score in patients with nonvalvular atrial fibrillation Clin Appl Thromb Hemost 2014 20 07 687 692 23430929 10.1177/1076029613478157

[26] Evans T C Jehle D The red blood cell distribution width J Emerg Med 1991 9 01 71 74 1955687 10.1016/0736-4679(91)90592-4

[27] Brækkan S K Hald E M Mathiesen E B Competing risk of atherosclerotic risk factors for arterial and venous thrombosis in a general population: the Tromso study Arterioscler Thromb Vasc Biol 2012 32 02 487 491 22075253 10.1161/ATVBAHA.111.237545

[28] Nyrnes A Mathiesen E B Njølstad I Wilsgaard T Løchen M L Palpitations are predictive of future atrial fibrillation. An 11-year follow-up of 22,815 men and women: the Tromsø Study Eur J Prev Cardiol 2013 20 05 729 736 22588086 10.1177/2047487312446562

[29] Braekkan S K Borch K H Mathiesen E B Njølstad I Wilsgaard T Hansen J B Body height and risk of venous thromboembolism: the Tromsø Study Am J Epidemiol 2010 171 10 1109 1115 20418276 10.1093/aje/kwq066

[30] WHO MONICA Project Principal Investigators The World Health Organization MONICA Project (monitoring trends and determinants in cardiovascular disease): a major international collaboration J Clin Epidemiol 1988 41 02 105 114 3335877 10.1016/0895-4356(88)90084-4

[31] Shao Q Korantzopoulos P Letsas K P Red blood cell distribution width as a predictor of atrial fibrillation J Clin Lab Anal 2018 32 05 e22378 29315856 10.1002/jcla.22378 PMC6817116

[32] Weiss G Goodnough L T Anemia of chronic disease N Engl J Med 2005 352 10 1011 1023 15758012 10.1056/NEJMra041809

[33] Ghaffari S Oxidative stress in the regulation of normal and neoplastic hematopoiesis Antioxid Redox Signal 2008 10 11 1923 1940 18707226 10.1089/ars.2008.2142 PMC2932538

[34] Neuman R B Bloom H L Shukrullah I Oxidative stress markers are associated with persistent atrial fibrillation Clin Chem 2007 53 09 1652 1657 17599958 10.1373/clinchem.2006.083923 PMC3158654

[35] Hu Y F Chen Y J Lin Y J Chen S A Inflammation and the pathogenesis of atrial fibrillation Nat Rev Cardiol 2015 12 04 230 243 25622848 10.1038/nrcardio.2015.2

[36] Lappegård J Ellingsen T S Hindberg K Impact of chronic inflammation, assessed by hs-CRP, on the association between red cell distribution width and arterial cardiovascular disease: the Tromsø Study TH Open 2018 2 02 e182 e189 31249941 10.1055/s-0038-1651523 PMC6524874

[37] Patel K V Mohanty J G Kanapuru B Hesdorffer C Ershler W B Rifkind J M Association of the red cell distribution width with red blood cell deformability Adv Exp Med Biol 2013 765 211 216 22879035 10.1007/978-1-4614-4989-8_29 PMC5939938

[38] Salvagno G L Sanchis-Gomar F Picanza A Lippi G Red blood cell distribution width: a simple parameter with multiple clinical applications Crit Rev Clin Lab Sci 2015 52 02 86 105 25535770 10.3109/10408363.2014.992064

[39] Hirayama T Roberts D Allers M Belboul A al-Khaja N William-Olsson G Association between arrhythmias and reduced red cell deformability following cardiopulmonary bypass Scand J Thorac Cardiovasc Surg 1988 22 02 179 180 3406694 10.3109/14017438809105954

[40] Lippi G Cervellin G Sanchis-Gomar F Red blood cell distribution width: a marker of anisocytosis potentially associated with atrial fibrillation World J Cardiol 2019 11 12 292 304 31908729 10.4330/wjc.v11.i12.292 PMC6937412

[41] Montagnana M Cervellin G Meschi T Lippi G The role of red blood cell distribution width in cardiovascular and thrombotic disorders Clin Chem Lab Med 2011 50 04 635 641 22505527 10.1515/cclm.2011.831

[42] Vayá A Suescun M Hemorheological parameters as independent predictors of venous thromboembolism Clin Hemorheol Microcirc 2013 53(1-2):131 141 22954636 10.3233/CH-2012-1581

[43] Yu F T Armstrong J K Tripette J Meiselman H J Cloutier G A local increase in red blood cell aggregation can trigger deep vein thrombosis: evidence based on quantitative cellular ultrasound imaging J Thromb Haemost 2011 9 03 481 488 21143377 10.1111/j.1538-7836.2010.04164.x PMC3050084

[44] Wan H Yang Y Zhu J The relationship between elevated red cell distribution width and long-term outcomes among patients with atrial fibrillation Clin Biochem 2015 48 12 762 767 26054582 10.1016/j.clinbiochem.2015.06.001

[45] Cha M J Lee H S Kim H M Jung J H Choi E K Oh S Association between red cell distribution width and thromboembolic events in patients with atrial fibrillation Eur J Intern Med 2017 46 41 46 28781193 10.1016/j.ejim.2017.07.028

[46] Malavasi V L Proietti M Spagni S Usefulness of red cells distribution width to predict worse outcomes in patients with atrial fibrillation Am J Cardiol 2019 124 10 1561 1567 31521256 10.1016/j.amjcard.2019.08.008

[47] Enga K F Rye-Holmboe I Hald E M Atrial fibrillation and future risk of venous thromboembolism: the Tromsø study J Thromb Haemost 2015 13 01 10 16 25330989 10.1111/jth.12762

[48] Hald E M Rinde L B Løchen M L Atrial fibrillation and cause-specific risks of pulmonary embolism and ischemic stroke J Am Heart Assoc 2018 7 03 e006502 29378729 10.1161/JAHA.117.006502 PMC5850231

[49] Glynn R J Rosner B Comparison of risk factors for the competing risks of coronary heart disease, stroke, and venous thromboembolism Am J Epidemiol 2005 162 10 975 982 16207808 10.1093/aje/kwi309

[50] Mahmoodi B K Cushman M Anne Næss I Association of traditional cardiovascular risk factors with venous thromboembolism: an individual participant data meta-analysis of prospective studies Circulation 2017 135 01 7 16 27831499 10.1161/CIRCULATIONAHA.116.024507 PMC5201424

[51] Rosendaal F R Venous thrombosis: a multicausal disease Lancet 1999 353(9159):1167 1173 10209995 10.1016/s0140-6736(98)10266-0

[52] Småbrekke B Rinde L B Hindberg K Atherosclerotic risk factors and risk of myocardial infarction and venous thromboembolism; time-fixed versus time-varying analyses. The Tromsø Study PLoS One 2016 11 09 e0163242 27635655 10.1371/journal.pone.0163242 PMC5026338

